# Rad51 and Rad52 Are Involved in Homologous Recombination of Replicating Herpes Simplex Virus DNA

**DOI:** 10.1371/journal.pone.0111584

**Published:** 2014-11-03

**Authors:** Ka-Wei Tang, Peter Norberg, Martin Holmudden, Per Elias, Jan-Åke Liljeqvist

**Affiliations:** 1 Department of Medical Biochemistry and Cell Biology, Institute of Biomedicine, The Sahlgrenska Academy, University of Gothenburg, Gothenburg, Sweden; 2 Department of Infectious Diseases, Section of Virology, Institute of Biomedicine, The Sahlgrenska Academy, University of Gothenburg, Gothenburg, Sweden; UC Irvine Medical Center, United States of America

## Abstract

Replication of herpes simplex virus 1 is coupled to recombination, but the molecular mechanisms underlying this process are poorly characterized. The role of Rad51 and Rad52 recombinases in viral recombination was examined in human fibroblast cells 1BR.3.N (wild type) and in GM16097 with replication defects caused by mutations in DNA ligase I. Intermolecular recombination between viruses, tsS and tsK, harboring genetic markers gave rise to ∼17% recombinants in both cell lines. Knock-down of Rad51 and Rad52 by siRNA reduced production of recombinants to 11% and 5%, respectively, in wild type cells and to 3% and 5%, respectively, in GM16097 cells. The results indicate a specific role for Rad51 and Rad52 in recombination of replicating herpes simplex virus 1 DNA. Mixed infections using clinical isolates with restriction enzyme polymorphisms in the US4 and US7 genes revealed recombination frequencies of 0.7%/kbp in wild type cells and 4%/kbp in GM16097 cells. Finally, tandem repeats in the US7 gene remained stable upon serial passage, indicating a high fidelity of recombination in infected cells.

## Introduction

**Figure 1:**
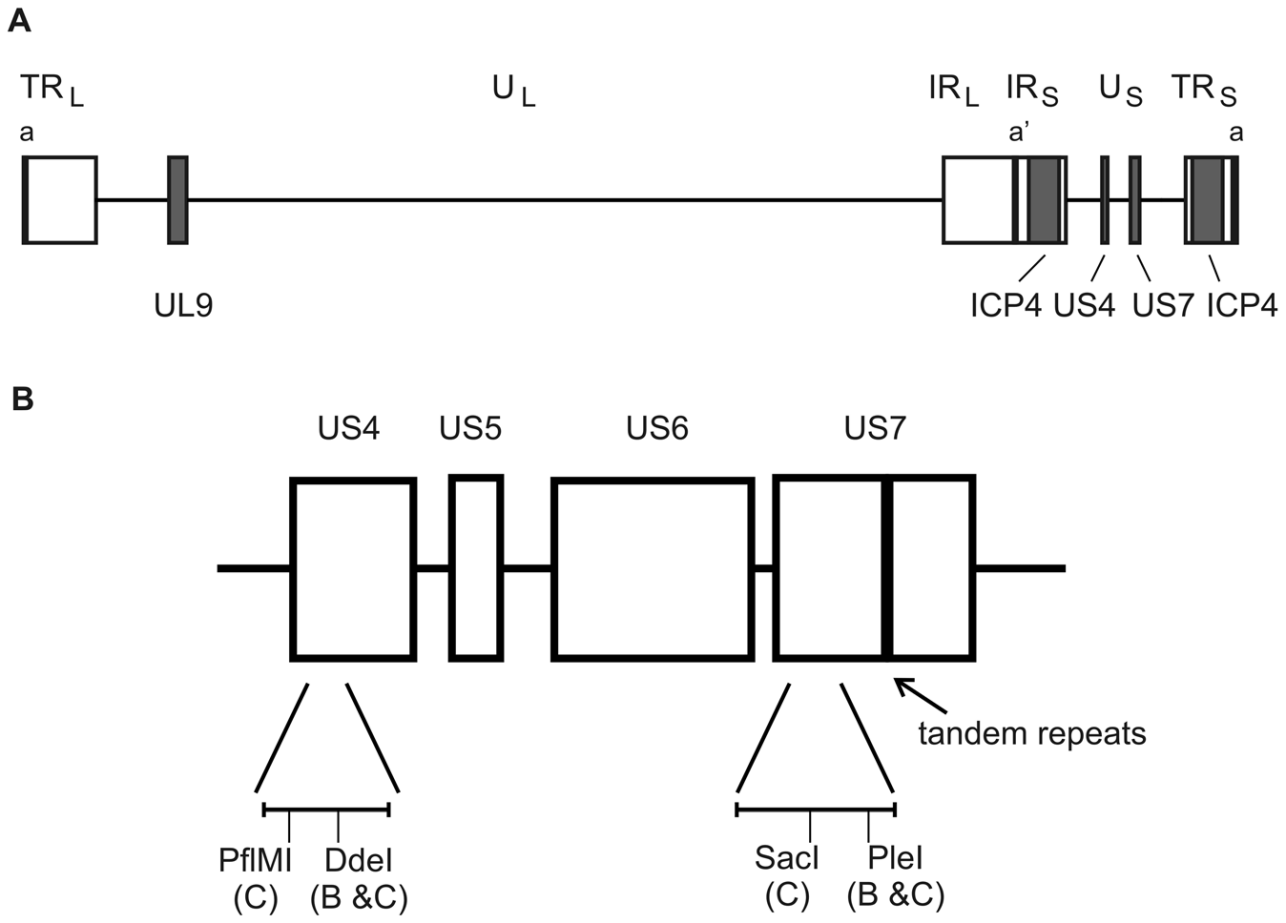
The HSV-1 genome and markers for recombination experiments. **A**, The bipartite HSV-1 genome. TR_L_ and TR_S_ denote terminal repeats and IR_L_ and IR_S_ are internal repeats [46]. The *a* sequence repeats (black bars) is located at the genome ends and in between the internal repeats. The tsS mutation is mapped to the UL9 gene and the tsK mutation is mapped to the ICP4 gene. A cluster of glycoprotein genes gG (US4) and gI (US7) in the U_S_ segment is used to examine recombination during serial propagation of virus. **B**, Enlargement of the glycoprotein gene cluster. The location of the 21 nucleotide tandem repeats is highlighted. The primer pair in the US4 gene generates a 269 bp PCR product. PflMI cleaves at nucleotide 55 in the PCR product for clade C, and DdeI cleaves at nucleotide 172 for clades B and C. The primer pair in US7 generates a 410 bp PCR product. SacI cleaves at nucleotide 222 for clade C and PleI at nucleotide 355 for clades B and C. The distance between the markers in US4 and US7 is approximately 3 kbp.

Homologous recombination is increasingly recognized as a major mechanism for maintaining genetic diversity of viruses. In fact, phylogenetic analyses of clinical herpes simplex virus 1 (HSV-1) isolates have shown that homologous recombination occurs frequently and that this mechanism seems to be essential for HSV-1 evolution, and for maintaining genomic integrity [1]–[6] Similar observations have also been made for other alphaherpesviruses [7]–[10]. Homologous recombination is also responsible for genomic isomerization occurring during HSV-1 replication [11]. The HSV-1 genome consists of two segments, designated unique long (U_L_) and unique short (U_S_). Each segment is flanked by inverted repeats containing a variable number of the *a* sequence (Figure **1A**). The *a* sequences are sites for cleavage of concatemeric genomes into monomers, which will be packaged into virus particles. During an infection a mixed population of genomes, in which the U_L_ and U_S_ segments exist in four different orientations, is created. The genome isomers are found in equimolar ratios, reflecting a high frequency of homologous recombination.

The importance of homologous recombination for HSV-1 to acquire genetic segments required for virulence has been demonstrated in animal models [12], [13]. Moreover, in vivo studies have shown high recombination rates between animal herpesviruses [14], [15].

After a primary infection of epithelial cells, HSV-1 establishes latency in sensory ganglia and intermittently reactivates, causing oral lesions. More commonly, however, the virus reactivates without symptoms i.e. asymptomatic shedding of virus. Several studies have shown that the same individual can be infected by different HSV-1 strains and that both original and recombinant strains can be reactivated [16]–[19]. As the HSV-1 infection is common, with a high prevalence worldwide, it is likely that different HSV-1 strains can replicate simultaneously in the same epithelial or nerve cell, making recombination between strains possible.

Homologous recombination is greatly enhanced by double-strand breaks in DNA, which can be repaired in an error-free manner employing Rad51, ensuring high-fidelity base-pairing between complementary strands [20]. During an HSV-1 infection homologous recombination may be initiated in several ways. Cleavage and processing of repeated *a* sequences take place between the inverted repeats of U_L_ and U_S_ segments, and may initiate homologous recombination, resulting in four possible isomers of the bi-partite genome. It has, in fact, been noted that *a* sequences themselves may enhance the frequency of homologous recombination approximately two-fold [21]. In addition, double-stranded breaks can be formed when the replication fork encounters a single-stranded break or gap. It is also possible that long stretches of single-stranded DNA exposed during replication might recruit the cellular recombinase Rad51 and initiate recombination. DNA damage caused by UV-irradiation has been shown to reduce viral replication, but increase recombination frequencies [22]. Moreover, knock-down of Rad51 and Rad52 by siRNA reduces viral replication of UV-irradiated HSV-1 150- and 100-fold respectively, indicating the important role of these recombinases during repair of replicating DNA [23]. Due to inherent properties of Rad51 the fidelity of homologous recombination is usually high, but the process is also controlled by mismatch repair that prevents recombination between related but non-identical molecules [24].

The HSV-1 genome contains several repeated sequence elements. The inverted repeats bordering the U_L_ and U_S_ segments are flanked by tandemly repeated sequences, *a* sequences, but repeated sequences can also be found within genes. One example is US7, encoding the HSV-1 glycoprotein I (gI) (Figure **1B**). This gene contains a tandem repeat region, and analysis of clinical HSV-1 isolates revealed that this region is polymorphic with 2–8 repeats encoding seven amino acids each [25]. The mechanism behind the emergence of directly repeated elements is likely to involve DNA replication and DNA repair pathways, and to result in the expansion or deletion of tandem repeats [26].

The molecular pathways underlining HSV-1 recombination are poorly characterized, but mounting evidence suggest that cellular repair and recombination pathways operate on virus DNA [2]. In addition, some viral proteins may have specific roles during recombination. For example, the single-strand DNA binding protein ICP8, may behave as a recombinase in vitro, and the alkaline 5′-3′exonuclease UL12 has also been implicated in virus recombination [27]–[29]. In fact, UL12 and ICP8 together can perform strand exchange *in vitro* [30]. UL12 stimulates recombination by a single-strand annealing mechanism on model substrates introduced into cellular DNA [31].

Here we have studied three different aspects of recombination occurring during HSV-1 replication. We examined the formation of recombinants during a single infectious cycle from tsK virus, with mutations in the ICP4 gene in the terminal repeats surrounding the U_S_ segment, and tsS virus, with a mutation in the UL9 gene in the U_L_ segment (Figure **1A**). We also investigated recombination between three clinical isolates during prolonged passage of virus by genotyping a region within the US4 and US7 genes (Figure **1B**). Finally, we examined the stability of a 21 nucleotide tandem repeat in the US7 gene after series of passages.

In order to delineate the molecular mechanisms underlying the different recombination events, the experiments were performed in human fibroblast cells 1BR.3.N (wild type) and GM16097. The latter cell line harbours mutations in DNA ligase I, preventing efficient sealing of nicks in replicated lagging strand DNA [32]. DNA ligase I has been shown to co-localize with viral replication proteins in intra-nuclear replication foci [33]. DNA ligase I has also been shown to participate in processing of lagging-strand intermediates *in vitro* together with HSV-1 DNA polymerase I and the Fen-1 nuclease [34]. A functional role for DNA ligase I during HSV-1 viral replication is also indicated by a small plaque phenotype for HSV-1 on GM16097 cells [35]. It was our assumption that reduced processing of lagging-strand intermediates would lead to enhanced recombination, thereby demonstrating a direct link between HSV-1 DNA replication and recombination. To identify the molecular pathway used for recombination, the contribution of the cellular recombinases Rad51 and Rad52 to viral recombination was assessed by knock-down experiments using siRNA.

## Results and Discussion

### Role of Rad51 and Rad52 in HSV-1 recombination

Existing results suggest a modest role for Rad51 during undisturbed DNA replication of the HSV-1 genome. Replication of UV-damaged viral DNA, however, is highly dependent on Rad51, Rad52 and Rad54 [23]. In addition, mismatch repair may also be involved in recombination of HSV-1 DNA [36]. Here, we examined the role of Rad51 and Rad52 during viral recombination in 1BR.3.N fibroblasts using mixed infections with two mutant strains, tsS and tsK, to generate wild type virus by recombination. These two viruses were derived from HSV-1 strain 17 [37]–[40]. Since the tsS mutation, which affects the UL9 origin-binding protein, maps in the U_L_ segment and the tsK mutation, which affects the transcription activator ICP4, maps in the terminal repeats surrounding the U_S_ segment of the HSV-1 genome, the assay may reflect *a* sequence-dependent recombination as well as general homologous recombination. In order to examine the coupling of recombination with DNA replication we also performed experiments in GM16097 cells containing mutations in the DNA ligase I gene.

**Figure 2:**
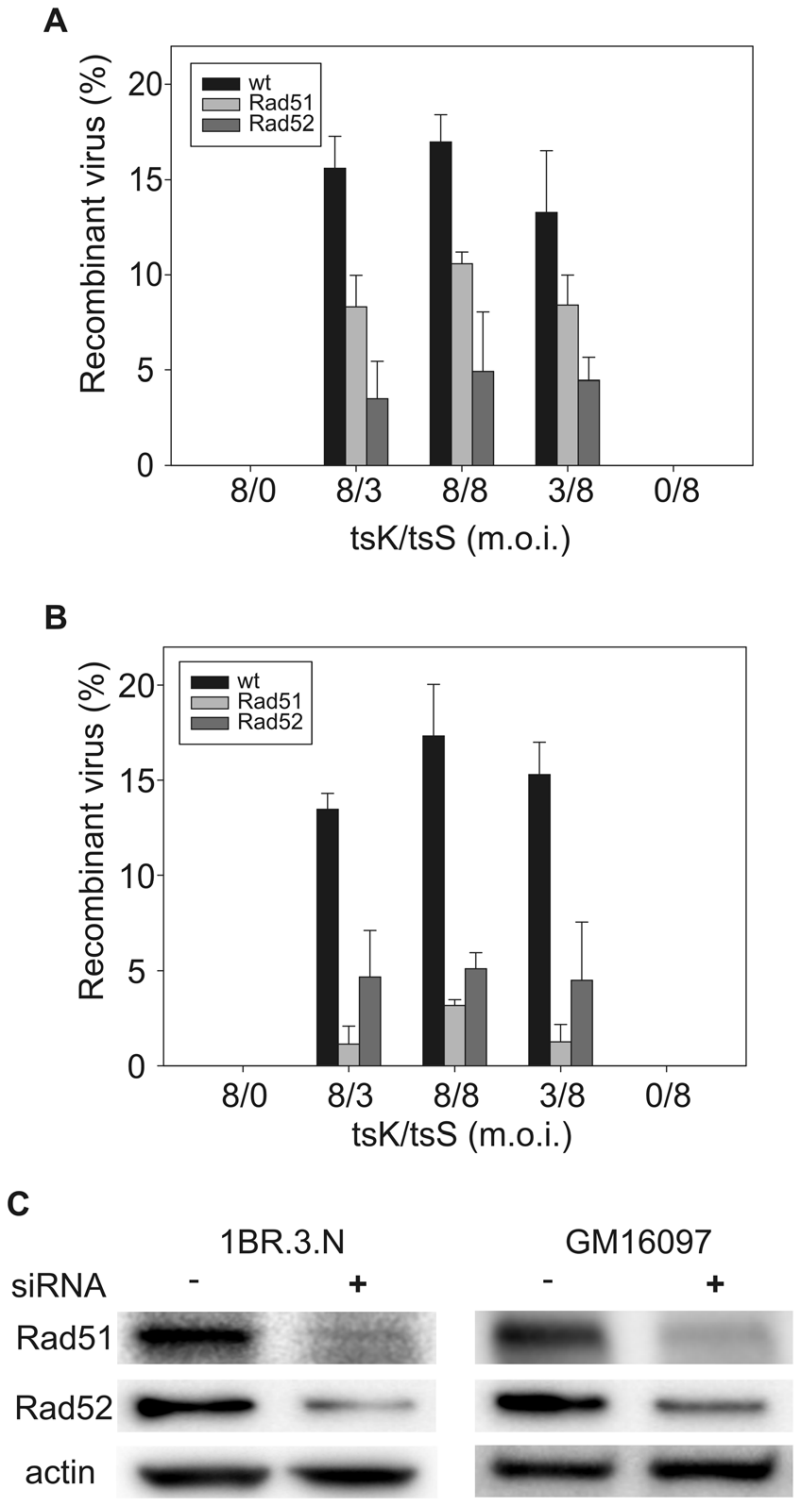
HSV-1 tsK and tsS recombination in 1BR.3.N and GM16097. **A**, Different mixtures of temperature-sensitive virus were added at the m.o.i. indicated to 1BR.3.N cells either mock-transfected or transfected with siRNA against Rad51 and Rad52. Percentage of recombinant virus was determined as the titer obtained by plaque assay on GMK-AH1 cells at 39°C (non-permissive temperature) divided by the titer obtained at 32**°**C (permissive temperature). Recombinant virus detectable in our assay contain the U_L_-segment (wt UL9 gene) from tsK virus and U_S_-segment (wt ICP4 gene) from tsS virus. Values represent average from three independent experiments and error bars indicate standard deviation. **B**, Recombination in GM16097 cells examined as described above. **C**, Western blots demonstrating knock-down of Rad51 and Rad52 proteins in 1BR.3.N and GM16097 cells.

Mixed infections were carried out at high multiplicity of infection (m.o.i.) using different ratios of the virus mutants at the permissive temperature (32°C). The experimental conditions were chosen in order to ensure that the infecting genomes were exposed to the same intracellular environment, and to minimize selection for recombinant virus during a second infectious cycle. The progeny of the infection would yield recombinants as well as non-recombinants. Virus was harvested 24 hours post-infection and virus titers were assayed at 32°C, a condition under which both the recombinant and non-recombinant virus can replicate efficiently, and at non-permissive temperature 39°C, a condition under which only the recombinants which have aquired a wild type genotype can replicate (i.e. the recombinant detectable in our assay containing the U_L_-segment (wt UL9 gene) from tsK virus and U_S_-segment (wt ICP4 gene) from tsS virus). The number of recombinants was calculated as the ratio of the virus titer at non-permissive temperature divided by the virus titer at the permissive temperature. The progeny detected at the non-permissive temperature represents at most one fourth of all possible intermolecular recombination events. The experiments were performed using different ratios of tsS and tsK virus, to demonstrate that the maximal yield of recombinants was obtained at equimolar ratios of the infecting strains. Very few/no revertants were detected in the experiments with only one viral strain at the non-permissive temperature. Even at an m.o.i. of 16, single ts-mutant infections yielded progeny with a titer less than 5×10^1^ pfu/ml in the non-permissive temperature (data not shown). Our results demonstrate efficient recombination in a single infectious cycle generating wild type recombinants from tsS and tsK virus (Figure **2A and 2B**). The frequency of recombinants, approximately 17% of total virus yield for both 1BR.3.N and for GM16097 cells, was high, approaching an expected maximal value of 25%. The results imply that intermolecular recombination during HSV-1 DNA replication is efficient and may account for a substantial fraction of isomerization of the U_L_ and U_S_ segments.

To investigate the genetic requirements for intermolecular recombination we performed siRNA knock-down experiments against the Rad51 recombinase. Under these conditions we observed that the yield of recombinants was reduced by 38% to a final yield of 11% in 1BR.3.N cells (P = 0.0042), and by 82% to a final yield of 3% in GM16097 cells (P = 0.0057) compared with mock-transfected cells. We also analyzed the role of Rad52. Here, we found similar effects on recombination. Knock-down of Rad52 gave 5% recombinants in both wild type cells (P = 0.0055) and DNA ligase I deficient cells (P = 0.0053) compared with mock-transfected cells. We also determined the efficiency of siRNA mediated knock-down by western blotting (Figure **2C**). In 1BR.3.N cells Rad51 was reduced to 23% and Rad52 to 29%. Corresponding values for GM16097 cells were 29% and 46%, respectively.

To ascertain the specificity of the siRNAs used we also performed experiments using a non-targeting siRNA (Table **1**). The results show a slight decrease in virus yield caused by siRNA against Rad51 and Rad52 (at 32°C P = 0.0124 and P = 0.0104 respectively, and at 39°C P = 0.003 and P = 0.004 respectively), but not by the non-targeting siRNA (at 32°C P = 0.1793, and at 39°C P = 0.4028) compared to mock-transfected cells. In the experiments with non-targeting siRNA, the yield of recombinant viruses was similar to that obtained in cells without siRNA (P = 0.1224). The decreased total yield of virus from cells treated with siRNA against Rad51 and Rad52 (P = 0.0007 and P = 0.0005 respectively) is likely caused by a reduced capability to repair double-stranded breaks formed during DNA synthesis, resulting in reduced formation of infectious virions as previously discussed [23]. The DNA synthesis machinery, however, is likely to operate independently of recombination until it meets a double-stranded break. We suggest that reduced recombination may cause a reduction in virus production.

**Table 1:** Effects of siRNA on replication and recombination.

siRNA	Titer at 32 (p.f.u./ml)	Titer at 39 (p.f.u./ml)	Recombinant virus (%)
None	1.9 (±0.5)×10^7^	3.2 (±0.6)×10^6^	17.3 (±1.4)
Rad51	6.3 (±3.2)×10^6^	5.3 (±2.5)×10^5^	8.6 (±1.3)
Rad52	5.2 (±2.7)×10^6^	3.6 (±1.7)×10^5^	7.1 (±1.4)
Non-targeting	1.5 (±0.3)×10^7^	3.0 (±0.7)×10^6^	20.0 (±2.9)

1BR.3.N cells were treated with lipids in the absence or presence of the siRNAs as indicated and subsequently infected by a mixture of tsK and tsS virus at m.o.i. 8. Recombinant virus detectable in our assay contain the U_L_-segment (wt UL9 gene) from tsK virus and U_S_-segment (wt ICP4 gene) from tsS virus. Values represent average from three independent experiments. Values in parenthesis indicate standard deviation.

We thus observe no difference in yield of recombinants between the non-transfected cell lines. In cells transfected with siRNA against Rad51, however, we observe a different outcome. This result may reflect varying success in reducing protein levels by siRNA in the two cell lines, but it may also indicate that more than one mechanism may exist for recombination during HSV-1 DNA replication. For example, recombination stimulated by the *a* sequence may be Rad51-independent, and occur by a single-strand annealing mechanism leading to loss of at least one repeat [20], [41], [42]. Indeed, both Rad52 and the HSV-1 single-strand DNA-binding protein ICP8 seem to assist UL12-mediated single-strand annealing in vivo [31]. The second alternative, general homologous recombination associated with DNA replication, would require Rad51. In 1BR.3.N cells Rad51 appears to contribute less to recombination in comparison with GM16097 cells. This observation may imply that *a* sequence-mediated recombination could generate a significant fraction of tsS/tsK recombinants, possibly by a single-strand annealing mechanism [31]. In contrast, recombination in GM16097 cells with a defect in DNA ligase I resulting in causing a defect in repair of lagging strand fragments appears to depend on Rad51 to a higher extent. We thus suggest that recombination caused by strand-breaks during DNA replication is Rad51 dependent.

In cells transfected with siRNA against Rad52 we observed a similar reduction of recombination in both cells lines. The possibility therefore exists that Rad52 may function both in recombination coupled to DNA replication and *a* sequence-dependent recombination as discussed above, but this remains a speculation.

Another possibility to join broken chromosomes utilizing imprecise non-homologous end-joining also exists, but the probability of generating viable virus genomes by random fusion of DNA ends from separate genomes would be very low. It is therefore a less likely mechanistic explanation for recombination coupled to replication.

To conclude, homologous recombination dependent on Rad51 and Rad52 contribute significantly to HSV-1 recombination and may, in fact, be the method of choice for recombination of replicating HSV-1 DNA.

### Recombination during serial passage of clinical isolates

Homologous recombination is characterized by high fidelity due to inherent properties of Rad51 combined with quality control contributed by mismatch repair [24], [36]. However, errors can still occur and the frequency of recombination and replication errors may be enhanced by the local DNA sequence. To study the fidelity of homologous recombination, we first examined recombination between genetic markers in US4 and US7, and subsequently the stability of a repeated sequence in the US7 gene. Recombination in the US4 and US7 region was measured during repeated passages in 1BR.3.N and GM16097 cells (Table **2**). We passaged three clinical HSV-1 isolates, classified as belonging to the previously described clades A, B and C, based on the nucleotide pattern in the US4 and US7 genes [3]. Using three isolates with distinct markers facilitates detection of recombinants. A sequence of 21 nucleotides in the US7 gene has been found to be repeated between 2–8 times in clinical isolates of HSV-1 [25]. The isolates belonging to clades A and B contain four repeats and the isolate belonging to clade C contains three repeats.

**Table 2:** Recombination during serial passage of HSV-1.

Cell line		Clade	Passage 1	Passage 5	Passage 10
1BR.3.N					
	Recombination (recombinants/total plaques)	A/B/C	2/94 = 2%	n.d.	7/79 = 9%
			1 A→C		2 A→C
			1 C→A		2 B→A
					3 C→A
	Composition of virus mixture	A	92%		86%
		B	3%		4%
		C	5%		10%
GM16097					
	Recombination (recombinants/total plaques)	A/B/C	11/93 = 12%	n.d.	19/63 = 30%
			4 A→C		7 A→C
			2 B→A		1 B→A
			4 C→A		4 B→C
			1 C→B		7 C→A
	Composition of virus mixture	A	58%		40%
		B	1%		8%
		C	41%		52%
**Viral titers (p.f.u./ml)**					
1BR.3.N		A/B/C	4.4×10^6^	1.4×10^6^	5.5×10^3^
		A	3.8×10^6^	2.5×10^6^	1.1×10^4^
		B	8.7×10^5^	3.5×10^5^	1.8×10^4^
		C	3.4×10^5^	1.7×10^5^	2.0×10^4^
GM16097		A/B/C	1.5×10^6^	1.6×10^5^	1.6×10^3^
		A	2.5×10^5^	6.3×10^4^	6.0×10^3^
		B	4.0×10^5^	8.0×10^3^	4.0×10^3^
		C	1.5×10^6^	9.7×10^4^	2.5×10^2^

* n.d.  =  not defined.

Three clinical HSV-1 isolates belonging to the different clades (A, B and C) were passaged separately or as a mixture ten times in 1BR.3.N or GM16097 cells. Recombination rate, type of recombination and percentage of each clade in the mixture are shown for passage 1 and passage 10. In total, 329 plaques from passage 1 and 10 were analyzed by a genotyping assay. The viral titers were determined for passage 1, passage 5 and passage 10.

The clinical isolates were passaged separately as well as a mixture of the A/B/C classified viruses. We noted that during repeated passages the total virus yield gradually declined for individual viruses as well as the mixture A/B/C (Table **2**). A similar observation has previously been described [43]. Although the behaviour of the different viruses are expected to be different during serial passage, the frequency of recombination during the first infectious cycle is likely to be unaffected by different growth properties. We observed a yield of 2% recombinants in 1BR.3.N cells and 12% in DNA ligase I defective GM16097 cells after a single passage. After 10 passages these numbers were 9% and 30%, respectively (Table **2**). The results appear to reflect an increased frequency of general homologous recombination dependent on a defect in DNA replication caused by impaired processing of Okazaki fragments formed during lagging stand replication [32].

As a control, we also analyzed the genotype for virus obtained from 25 plaques for each of the clinical isolates. All plaque-purified viruses derived from a single isolate showed the expected genotype. It is thus unlikely that selection based on pre-existing genetic heterogeneity can explain our results (data not shown).

The distance between recombination markers in US4 and US7 is approximately 3 kbp, and we can therefore calculate a recombination frequency of 0.7%/kbp in 1BR.3.N cells and 4%/kbp in GM16097 cells. These values most likely relate to a single recombination event. Corresponding values for tsS/tsK recombination would be 0.22%/kbp and 0.24%/kbp, assuming average distance between the markers in the U_L_ and U_S_ segments to be 85 kbp. A prerequisite for the detection of a recombination event by analyzing two loci is that the genotypes of these differ and that the recombinant genome contains one locus from each of the two parental strains. An even number of recombination crossovers between the loci will leave the recombination event undetectable. A consequence is that the detectable recombinant frequency will at most be close to 50% even if all strains are recombinants since only recombinants with an odd number of recombination crossovers can be detected. In our tsK/tsS recombination-assay we will only be able to detect the recombinant virus containing the wild type UL9 and ICP4 genes, i.e. U_L_-segment from tsK-virus and U_S_-segment from tsS-virus, which could be generated by genomic isomerization. Since the two loci are located in U_L_ and U_S_, respectively, the equimolar portions generated will average out the distance, and the exact locations of the loci are therefore irrelevant. Because only 4 out of 16 possible isomers contain the wild type markers mentioned above the expected detection rate will be 25%. The recombination-assay can also detect a general homologous recombination, for which recombination will increase approximately proportionally with the distance between these loci, which for UL9 and the nearest ICP4 is approximately 24 kbp or 108 kbp, depending on isomer. It is therefore likely that the frequency of tsS/tsK recombination presented here is underestimated. In previous studies recombination frequencies range between 0.26%/kbp and 3.2%/kbp [44]. The highest value obtained comes from a study in which recombination has been enhanced by UV-irradiation, and we know now that Rad51-dependent homologus recombination is directly involved in repair of UV-damaged HSV-1 DNA [22], [23].

We also noted a selection and/or random fluctuation of populations during serial passage of the A/B/C mixture. After one passage in 1BR.3.N cells, clade A made up 92% of total plaques analyzed. Clades B and C were 3% and 5%, respectively. Two percent of all plaques were recombinants of type A and C recombination. After ten passages clade A constituted 86%, clade B 4% and clade C 10%. Nine percent were found to be recombinants. For GM16097 cells, after one passage clade A constituted 58%, clade B 1% and clade C 41%, while 12% were classified as recombinants. After ten passages clade A constituted 40%, clade B 8% and clade C 52%, and 30% of the progeny were recombinants. In accordance with the distribution frequency most recombinants were formed between the A and C clades (78% and 73% in respective cell line).

The uneven distribution of HSV-1 from different clades reduced the possibility to detect recombinants because recombination between viruses belonging to the same clade could not be detected in our system. The recombination frequency is therefore probably underestimated, especially in the 1BR.3.N cells. As homologous recombination is facilitated by high replication [45] and the titers already decreased 10-fold at passage 5 in the GM16097 cells, the number of recombinants at passage 10 should probably increase significantly if the HSV-1 strains could replicate to the same extent as in the control cell line. These considerations support the notion that a defect in DNA ligase I causes diminished capacity to repair strand-breaks thereby enhancing homologous recombination.

The fidelity of recombination could be examined by investigation of the stability of the repeated sequences, since replication errors processed by recombination might generate genetic variability. Analysis of the number of tandem repeats was carried out for 20 plaques from each cell line after 10 passages, using a length-specific PCR method [3]. The number of repeats did not change in any of the clinical isolates used during serial passage, and in the recombinant isolates the number of repeats was the same as for the parental strains (data not shown). To confirm this observation and to compensate for the titer decrease of the clinical isolates we used a single laboratory strain with five copies of tandem repeats in US7, which we infected at a low m.o.i. (one tenth of the amount used in the previous experiment), incubated until full complete cytopathic effect was observed (5–6 days) and passaged 25 times. The virus pool of every fifth passage showed no change in the number of tandem repeats (data not shown). We thus conclude that, despite a high frequency of recombination combined with a defect in processing of replication intermediates, we could not detect variations in the number of repeats. Our observation implies that recombination is characterized by high fidelity, and that the nucleotide sequence in the repeat region of the US7 gene is unlikely to have properties enhancing errors during DNA replication, recombination and repair.

Systematic compilations of tandemly repeated sequences in the HSV-1 genome have been published [6], [46]. The most well-known example concerns the terminal *a* sequence repeats, but repeats also exist in coding sequences. For example, the gene for ICP34.5 contains a sequence of 9 nucleotides repeated 10 times in the F strain but only 5 times in the 17syn+ strain [47]. The lengths of variable-number tandem repeats, VNTRs, are difficult to determine accurately with current rapid sequencing techniques, but in a number of cases it has been demonstrated that repeat lengths vary not only between strains but also during multiple passages of the same strain [48]–[50]. The stability of the various classes appears to differ but a mechanistic explanation of these phenomena is currently lacking. It is worth noting that Rad51-dependent homologous recombination is likely to preserve the number of repeats, whereas recombination by a single-strand annealing mechanism will cause deletions of repeated sequences and intervening sequences. It is thus possible that *a* sequence-mediated recombination may to some extent involve single-strand annealing generating variable numbers of repeats, whereas recombination within the U_S_ and U_L_ segments is likely to rely on Rad51 to more faithfully preserve genetic integrity.

## Conclusions

We have demonstrated that Rad51 and Rad52 dependent homologous recombination is coupled to HSV-1 DNA replication. Other mechanisms, e.g. single-strand annealing, may also participate during *a* sequence-mediated isomerization of the U_L_ and U_S_ segments of the virus genome. Biochemical evidence support a role of viral proteins such as ICP8/UL29 and the alkaline exonuclease UL12 in HSV-1 recombination, but it remains to be demonstrated that ICP8 has a specific role in recombination in infected cells separate from its role in DNA replication.

Recombination during an infectious cycle appears to be an efficient mechanism to sustain genetic competence for viral genomes and repair of damaged DNA, and as described in this study, the fidelity of recombination appears to be high since the tandem repeats in the US7 gene remain stable. This observation lends support to studies arguing that measurements of repeats can be used to monitor transmission and pathogenesis of HSV-1 infections [48]. The molecular mechanisms causing existing variation in repeats between different strains has yet to be characterized.

Finally, HSV-1 recombination offers a readily accessible experimental system for studying mechanisms of recombination in human cells.

## Materials and Methods

### Cells, viruses and antibodies

1BR.3.N human fibroblasts (ECACC 90020508) were used as control cells and propagated in Dulbecco's modified Eagle medium supplemented with 10% fetal bovine serum and non-essential amino acids (GIBCO BRL). The GM16097 human fibroblast cell-line (CCR repository number GM16097) has mutations in both the paternal (E566K) and maternal (R771W) allele of DNA ligase I gene, resulting in a retarded ligation of Okazaki-fragments and aberrant DNA repair [51]. The cells were grown in Dulbecco's modified Eagle medium containing 10% fetal bovine serum and non-essential amino acids. African green monkey kidney (GMK-AH1) cells were obtained from the Swedish Institute for Infectious Disease Control, Stockholm and cultivated in Eagle's minimum essential medium supplemented with 2% fetal calf serum, 0.05% Primaton RL (Kraft Inc., Norwich, CT), 100 U/ml of penicillin, and 100 µg/ml of streptomycin.

The HSV-1 temperature-sensitive mutant viruses, both derived from strain 17 where: tsK, an A475V mutant defective in ICP4, the major immediate early transcriptional regulator, and tsS, an A90T mutant defective in UL9/OBP, the origin binding protein. Three clinical HSV-1 isolates, all derived from oral lesions and obtained at the Department of Infectious Diseases, Section of Virology, University of Gothenburg, Sweden, were included in this study. These isolates; 99–3515 (clade A, four tandem repeats in US7), 90–395 (clade B, four tandem repeats in US7) and 94–783 (clade C, three tandem repeats in US7) were classified as non-recombinants based on DNA sequencing of the genes coding for US4 and US7 genes. Viral stocks were prepared by infecting GMK-AH1 cells cultured in Eagle's minimal essential medium supplemented with 2% calf serum and antibiotics.

Antibodies against beta-actin (ab6276), Rad51 (ab213) and Rad52 (ab18264) were purchased from Abcam.

### tsK/tsS recombination

Viruses were mixed at specified ratios and used to infect 1BR.3.N or GM16097 cells in 6-well plates at the permissive temperature (32°C). Twenty-four hours post-infection the supernatant was harvested and cleared by centrifugation. The virus yields were subsequently titrated on GMK-AH1 cells at permissive (32°C) and non-permissive (39°C) temperature. Recombination was calculated as the virus titer at non-permissive temperature divided by the virus titer at permissive temperature [52].

### siRNA knock-down

Validated siRNAs Hs_RAD51_7 and FlexiTube siRNAs Hs_RAD52_5, Hs_RAD52_6, Hs_RAD52_7 were purchased from Qiagen. ON-TARGETplus Non-targeting Pool D-001810-10-20 were purchased from Dharmacon. Cell monolayers in 12-well plates were transfected with 80 pmol of siRNA, or a pool containing 20 pmol of each siRNA duplexes respectively by using Oligofectamine (Invitrogen). Mock-transfected cells were treated with Oligofectamine only. Cells were incubated for 72 hours and infected as above.

Following infection cells were washed with PBS and lysed in a buffer containing 0.5% SDS, 50 mM Tris-HCl pH 7.5, 150 mM NaCl, 1 mM EDTA, 25 mM DTT and 1X Protease inhibitor (Roche). Samples were further processed by passing the lysate through a syringe ten times. Samples were mixed with equal volumes of Laemmli's buffer (Bio-Rad) supplemented with 1/10 volume beta-mercaptoethanol. Samples were boiled for 5 minutes and subjected to electrophoresis on a 10% bisacrylamide gel. The gel was subjected to western blotting by transfer to PVDF-membranes, and subsequently blocked for 1 hour in PBS with 5% milk. The appropriate primary antibody diluted in PBS was added and incubated in 0.05% Tween and 5% milk for 1 hour. The membrane was then washed six times in PBS with 0.05% Tween and reacted with the appropriate secondary antibody for 1 hour diluted in PBS with 0.05% Tween and 5% milk. Finally, the membrane was washed six times in PBS with 0.05% Tween. The blots were developed by incubating membrane in Immun-Star WesternC (Bio-Rad) and quantified using Chemidoc XRS.

### Passage of clinical isolates

Equal amounts of virus from each of the three isolates were mixed and 500 µl of the mixture was added in duplicate, at an m.o.i. of 7, to 1BR.3.N cells or GM16097 cells. The same amount of viruses from each isolate was added into a separate well as control. After 1 hour incubation at 37°C the cells were washed gently three times and 2 ml medium was added. When complete cytopathic effect was observed (48 hours), cells and medium were harvested and freeze-thawed once at −70°C followed by centrifugation in an Eppendorf centrifuge for 10 minutes at 2000× *g*. Five hundred microliters of the supernatant was further passaged onto non-infected cells. This passage procedure was repeated 10 times. Viruses from each passage were stored at −70°C.

### Detection of recombinants and tandem repeats

To investigate the distribution of the different HSV-1 isolates within the virus mixture from different passages, 500 µl of the cell culture supernatant was first diluted to approximately 100 plaque forming units/well and incubated together with GMK-AH1 cells for 1 hour with addition of methylcellulose solution. In total, 187 plaques from passage 1 and 142 plaques from passage 10 from the wild type cells and the GM16097 cells were collected and viruses were transferred into GMK-AH1 cell tubes. After observing complete cytopathic effect (five to seven days), the virus-DNA was extracted in a Magnapure LC robot (Roche Diagnostics, Mannheim, Germany) using the Magnapure DNA Isolation Kit according to the manufacturer's instructions. Clade designation and homologous recombination between the US4 and US7 genes were detected by a genotyping technique based on amplification of the selected segments of the US4 and US7 genes by PCR, followed by restriction enzyme cleavage using specific sites for respective genotype [53]. As expected, the plaques showed cleavage patterns belonging to one clone only. In order to verify the assay, we also sequenced recombinants and non-recombinants and the results were in perfect agreement with the restriction site polymorphism assay (data not shown).

Analysis of the number of the tandem repeats was carried out for 20 plaques from each cell line after 10 passages, using a length-specific PCR method [3].

### Statistical analysis

P-values were calculated by using a one-tailed t-test with unequal variance, (Welch's t-test), and values of P<0.05 were regarded as significant. The test was chosen for its suitability for comparison of small data sets that are independent and assumed to be normally distributed. Welch's t-test was opted for over Student's t-test, due to the possibility of heteroscedasticity.
